# Anti-oral cancer properties of potential probiotic lactobacilli isolated from traditional milk, cheese, and yogurt

**DOI:** 10.1038/s41598-024-57024-y

**Published:** 2024-03-16

**Authors:** Yousef Nami, Omid Tavallaei, Amir Kiani, Nesa Moazami, Mahya Samari, Hossein Derakhshankhah, Mehdi Jaymand, Babak Haghshenas

**Affiliations:** 1 Department of Food Biotechnology, Branch for Northwest and West Region, Agricultural Biotechnology Research Institute of Iran, Agricultural Research, Education and Extension Organization (AREEO), Tabriz, Iran; 2 Pharmaceutical Sciences Research Center, Health Institute, School of Pharmacy, Kermanshah University of Medical Sciences, Kermanshah, Iran; 3 Regenerative Medicine Research Center (RMRC), Health Technology Institute, Kermanshah University of Medical Sciences, Kermanshah, Iran; 4 Students Research Committee, Faculty of Pharmacy, Kermanshah University of Medical Sciences, Kermanshah, Iran; 5 Department of Applied Chemistry, Faculty of Chemistry, Razi University, Kermanshah, Iran; 6 USERN Office, Kermanshah University of Medical Sciences, Kermanshah, Iran; 7 Nano Drug Delivery Research Center, Health Technology Institute, Kermanshah University of Medical Sciences, Kermanshah, Iran

**Keywords:** *Lactobacillus*, Oral cancer, Probiotics, Apoptosis, Anti-cancer, Cancer, Microbiology

## Abstract

This study investigates the probiotic and anti-cancer effects of 21 isolated *Lactobacillus* strains from cheese, milk, and yogurt in Kermanshah, Iran, on oral cancer cell lines KB and OSCC. Four selected isolates (Y33, M45, C5, and C28) displayed good viability and resistance to specific antibiotics. Notably, strains C28 and Y33 exhibited the best results, showing susceptibility or semi-susceptibility to five antibiotics. Y33, with high cell surface hydrophobicity (62%), demonstrated significant anti-pathogenic activity, inhibiting the growth of tested pathogens and displaying strong adhesion to human intestinal Caco-2 cells (52%). Further assessments, including acridine orange/ethidium bromide staining and mRNA expression analysis, revealed four isolates (C5, C28, M45, and Y33) with promising probiotic properties. Particularly, Y33’s protein-based extract metabolites showed dose- and time-dependent inhibition of KB and OSCC cancer cell lines, inducing apoptosis without significant cytotoxic effects on normal cells. Y33 (*Lactiplantibacillus plantarum*) exhibited the strongest probiotic potential, surpassing conventional anti-cancer drugs, suggesting its therapeutic potential for preventing oral cancer cell proliferation and improving survival rates in oral cancer patients.

## Introduction

Probiotic bacteria are increasingly acknowledged for their health-promoting properties and therapeutic potential. Their significant anti-cancer capabilities are particularly noteworthy, as demonstrated in recent studies, showcasing the efficacy of specific probiotic strains, especially those derived from traditional dairy products, in inducing apoptosis and inhibiting proliferation in various cancer cell lines^1^.

Oral cancer stands as a prevalent malignancy, with an annual mortality rate of 177,757 cases and 377,713 new cases diagnosed worldwide each year. The highest incidence rates are observed in South Central Asia and India. The etiology of oral cancer is multifactorial, with major contributing factors including dietary elements, infections, radiation exposure, papillomavirus, smoking, alcohol consumption, and genetics. Consequently, the reduction of alcohol consumption and smoking plays a crucial role in mitigating the risk of oral cancer^2^.

Oral squamous cell carcinoma (OSCC), accounting for over 90% of cases, manifests with a diverse range of clinical symptoms. Despite advancements in treatment methods for oral cancer, there has been only a slight improvement in the overall survival rate for cancer patients in recent decades. Therefore, the development of novel and practical approaches, such as probiotic treatment, is deemed necessary^3^.

Probiotics, classified as beneficial, contribute to host health in various ways. Criteria for selecting probiotics encompass tolerance to gastrointestinal conditions, including bile acid and salt, safety, ability to adhere and colonize the intestinal tract, and promotion of host health^4^. The promising outcomes from these studies suggest that probiotic treatment could serve as an alternative to more invasive cancer treatments like chemotherapy and radiotherapy.

According to scientific research, probiotics, particularly *Lactobacillus* strains, have been found to have anticancer effects. Specifically, *Lactobacillus* strains have been shown to have antitumor activity by inducing apoptosis, inhibiting angiogenesis, and modulating the immune system^3^. However, it is important to note that the evidence for the anticancer effects of probiotics is still limited, and more research is needed to establish their efficacy. Furthermore, the effects of probiotics on cancer may vary depending on the type of cancer, the stage of cancer, and the type of probiotic used. On the other hand, while probiotics, especially *Lactobacillus* strains, have shown promise in their anticancer effects, more research is needed to establish their efficacy and safety^4^. In vitro evaluations are commonly employed to investigate and compare the anti-cancer properties of medicinal substances, including probiotics, due to challenges associated with studying in vivo properties. Cell death primarily occurs through apoptosis and necrosis, each having distinct biochemical and morphological events leading to cell demise. Apoptosis induction is important in cancer therapy because it is a mechanism that triggers the death of cancer cells. In most cells, certain proteins known as “caspases” trigger apoptosis, which is especially important for the treatment of cancer, since inducing cell death in cancer cells can help in their elimination. Cancer cells typically have an elevated threshold for endogenous pro-apoptotic signals, which can lead to a dangerously extended cellular life span and progressively malignant behavior. Conventional cancer treatment, such as chemotherapy and/or ionizing radiation therapy, overcomes apoptosis resistance by inducing extensive and indiscriminate damage in all rapidly dividing cell types, including many normal cell types^5^. However, this approach can cause severe side effects. Therefore, researchers have focused on the design of new strategies that more selectively tip the balance of cellular fate of cancer cells toward apoptosis while sparing normal cells^6^. There is a established correlation between the anti-cancer activity of therapeutic agents and apoptosis in various cancer cell lines. Diverse methods exist to evaluate apoptosis, ranging from biochemistry and simple light microscopy to advanced techniques like flow cytometry. Doxorubicin and paclitaxel are two chemotherapy drugs that are commonly used in cancer therapy. They are often used in combination with other drugs to treat various types of cancer, including breast cancer, ovarian cancer, and non-small cell lung cancer. Doxorubicin is an anthracycline antibiotic that works by inhibiting the activity of an enzyme called topoisomerase II, which is involved in DNA replication and repair. By inhibiting this enzyme, doxorubicin prevents cancer cells from dividing and multiplying. Paclitaxel, on the other hand, is a taxane that works by stabilizing microtubules, which are structures that help move chromosomes during cell division. By stabilizing microtubules, paclitaxel prevents cancer cells from dividing and multiplying. It is important to note that doxorubicin and paclitaxel, like other chemotherapy drugs, can cause side effects, such as nausea, vomiting, hair loss, and fatigue. However, the benefits of these drugs in treating cancer often outweigh the risks of side effects^7–9^. This study seeks to explore the isolated probiotic properties of *Lactobacillus* strains derived from commonly consumed traditional dairy products in Kermanshah, including cheese, milk, and yogurt. Additionally, the research assesses the impact of extracted metabolites from selected Lactobacillus strains on human oral cancer cell lines (KB and OSCC) and normal cell lines (fibroblast and HUVEK). The evaluation involves determining IC_50_ values through the MTT assay, and the obtained data is compared with both drug-treated (doxorubicin and paclitaxel) and untreated cells.

The anti-cancer mechanisms were characterized by assessing a combination of typically treated cell lines. Additionally, apoptosis morphological characteristics, such as membrane blebbing, membrane integrity, and nucleus fragmentation, were evaluated using fluorescent staining and the analysis of mRNA expression related to apoptosis-related genes. In vitro evaluations, including the release of lactate dehydrogenase assay, glutathione depletion assay, MTT assay, and macromolecular synthesis, were employed to investigate and compare the anti-cancer properties of probiotics. Apoptosis and necrosis, the two primary forms of cell death, were examined using various methods, ranging from biochemistry and simple light microscopy to sophisticated techniques such as flow cytometry. Nevertheless, the gold standard in apoptosis analysis remains the examination of morphological characteristics during cell death through acridine orange/ethidium bromide (AO/EB) staining and fluorescent microscopy.

## Material and method

### Isolation and culturing of *Lactobacillus* strains

A total of 150 dairy samples, including traditional cheese, milk, and yogurt, were utilized for the isolation of *Lactobacillus* strains. These samples were randomly collected from various rural areas in Kermanshah Province, Iran. To facilitate the isolation and separation of bacterial strains from the dairy products, one gram of each sample was dissolved in 9 mL of sterile trisodium citrate, followed by one-hour incubation at 4 °C. Subsequently, 1 mL of the solution was added to 10 mL of MRS culture medium. The cultures were then grown anaerobically at 37 °C overnight, followed by propagation in an MRS agar medium under the same conditions. Basic morphological and biochemical tests, including morphological cell analysis, catalase test, and gram staining, were employed for the identification and isolation of bacterial colonies^4^.

### Simulated digestive conditions tolerance test

To assess the tolerance of the isolated *Lactobacillus* strains to adverse conditions, a 10 mL sample of each bacterial culture was harvested after 24 h and then centrifuged at 5000×*g* for 4 min. Following the removal of the supernatant, the pelleted cells were re-suspended in 10 mL of MRS medium adjusted to pH 2.5 (oral pH) and 6.6 (low pH), as well as in 10 mL of growth medium containing 0.3% w/v ox gall and adjusted to pH 6.8. The cells were gently agitated for 3 h and 4 h, respectively, under anaerobic conditions at 37 °C.

The optical density (OD) was measured using a spectrophotometer at 600 nm to identify bacterial strains with higher tolerance to the tested conditions, following the methodology outlined by Yang and colleagues^7^. The OD of the control and treated strains was compared, and bacterial survival rates (%) were estimated using the following formula:

$$ Survival\;percentage = \frac{{\left[ {OD} \right]after\;treatment}}{{\left[ {OD} \right]befor\;treatmrnt}} \times 100 $$

Here OD treated is the treated sample, and OD control is the control optical density.

To mimic the conditions of the digestive tract, the culture medium of the selected strains underwent two distinct treatments. Firstly, to simulate the gastric environment, pepsin was added to the medium at a concentration of 5% (w/v) at pH 2.5, followed by incubation at 37 °C (110×*g*, 2 h). Secondly, to simulate the intestinal environment, a solution containing bile salts and pancreatin (0.3% and 0.1% (w/v), respectively) was added to the MRS medium at pH 6.0 and incubated at 37 °C (110×*g*, 3 h). Subsequently, bacterial cells were cultured on MRS agar medium for 48 h at 37 °C, and the number of growth colonies was counted^7^. The survival rate of the bacterial strains in simulated digestive conditions was then calculated using the following equation:

$$ Survival\;percentage = \frac{logCFU\;N1}{{logCFU\;N0}} \times 100 $$

where N_1_ indicates the total number of bacteria after treatment with harsh conditions, and N_0_ indicates the total number of bacteria before treatment.

### Anti-pathogen activities

The disc diffusion technique was utilized to assess the antagonistic activities of the isolated strains against various common human pathogens. The targeted pathogens included *Streptococcus sanguinis* PTCC 1449, *Yersinia enterocolitica* ATCC 23715, *Streptococcus salivarius* PTCC 1448, *Streptococcus sobrinus* PTCC 1601, *Listeria monocytogenes* ATCC 13932, *Streptococcus mutans* PTCC 1683, *Pseudomonas aeruginosa* PTCC 1181, *Staphylococcus aureus* ATCC 25923, *Bacillus subtilis* ATCC 19652, *Shigella flexneri* PTCC 1234, and *Klebsiella pneumoniae* PTCC 1053.

The agar well diffusion method involves creating wells in the agar medium inoculated with the target pathogen and introducing the test substance into these wells. The antimicrobial compounds diffuse from the well into the surrounding medium, inhibiting the growth of the pathogen and forming a clear inhibition zone around the well. Measurement of the inhibition zone size provides an indication of the effectiveness of the test substance against the pathogen. This method is relatively straightforward, offering a qualitative assessment of the antimicrobial activity. It’s important to note that results may be influenced by factors such as the diffusion rate of the test substance, the thickness of the agar medium, and the inoculum density of the pathogen^7^. Therefore, it is advisable to include appropriate controls and conduct experiments in triplicate to ensure the reproducibility of results.

### Antibiotics sensitivities

The antibiotic susceptibility of the bacterial strains was determined using the agar diffusion technique, employing commonly used antibiotics, including ciprofloxacin (5 μg), doxycycline (30 μg), cefixime (5 μg), amoxicillin (25 μg), azithromycin (15 μg), trimethoprim-sulfamethoxazole (75 μg), amoxicillin–clavulanic acid (10 μg), cephalexin (30 μg), and vancomycin (30 μg). The bacterial strains were cultured on MRS agar at 37 °C overnight, and antibiotic discs were then placed on the inoculated agar medium^8^.

### Probiotic characterization

#### Cell surface hydrophobicity analyze

To assess the hydrophobicity of the cell surface, bacterial strains were cultured overnight and subsequently centrifuged at 6000×*g* for 10 min. The resulting precipitated cells were then re-suspended in PBS (3 mL) to achieve a concentration of 1 × 10^8^ CFU/mL. The initial absorbance was measured at 600 nm (A0), and the bacterial suspension was vortexed before being treated with 1 mL of xylene (Merck, Germany) for 2 min^7^. After separation of the phases at 37 °C, the absorbance of the aqueous phase was measured (A1). Finally, the hydrophobicity of the cell surface was determined as a percentage using the following equation:

$$ Cell\;surface\;hydrophobicity\left( \% \right) = 1 - \frac{A1}{{A0}} \times 100 $$

This technique was applied to examine the bacterial cell surface hydrophobicity of the studied strains.

#### Cell adhesion assay

For the cell adhesion assay, CaCO_2_ cells were cultured on glass slides in tissue culture plates with RPMI-1640 (Sigma), supplemented with 10% fetal bovine serum and 50 units/mL penicillin–streptomycin. The monolayers were washed twice with sterile PBS (pH 7.4), and 10 mL of bacterial suspension (1 × 10^7^ CFU/mL) was added to each plate. The cell plates were then incubated, and non-adherent bacterial cells were removed by washing the plates three times with PBS^7^.

To isolate adherent bacterial cells, a trypsin–EDTA solution (0.05%) was employed, and the cells were suspended in 10 mL saline solution. The bacteria were cultured on MRS agar at 37 °C for 24 h. The adhesion percentage was calculated by comparing the number of attached bacterial cells to the total number of bacterial cells.

#### Cholesterol absorption test

To assess the ability to absorb cholesterol, bacterial strains were cultured in MRS medium at 37 °C, which included 0.3% bile salt (oxgall bile) and 150 μg/mL water-soluble cholesterol (poly-oxyethyl cholesteryl sebacate; Sigma). Subsequently, the cells underwent centrifugation at a rate of 4000×*g*. The quantity of cholesterol present in the aqueous phase was determined using the o-phthaldehyde method, ensuring the integrity of the analysis without any plagiarism^7^.

#### Hemolytic activity

After overnight culturing of bacterial cells on Oxoid blood agar, which incorporates 7% (v/v) sheep blood and is incubated for 48 h at 37 °C, three distinct classifications were observed to assess hemolytic activity. These classifications include the presence of light halos surrounding bacterial colonies for β-hemolysis, a light halo around the colonies for α-hemolysis, and the absence of a halo around the colonies for γ-hemolysis^9^.

#### Auto-aggregation and co-aggregation abilities

To perform auto-aggregation analysis, strains were harvested after 24 h and incubated at a concentration of 1 × 10^9^ CFU/mL, followed by centrifugation at 4000×*g* for 10 min. The cells were then washed and suspended in sterile PBS, maintaining a temperature of 37 °C for 4 h. Co-aggregation can be calculated using the equation below^8^:

$$ {\text{Co - aggregation}}\left( {\text{\% }} \right) = \frac{{\left( {A0 - At} \right)}}{At} \times 100 $$

where A_0_ demonstrates adsorption at time 0 and A_t_ represents adsorption at time t.

Meanwhile, for the co-aggregation test, equal volumes of different bacterial strains and three pathogens, namely *E. coli, L. monocytogenes*, and *B. subtilis*, were incubated for 4 h at 37 °C without agitation. The co-aggregation percentage was determined using the following equation:

$$ {\text{Co - aggregation}}\left( {\text{\% }} \right) = \left[ {\frac{{\left( {Ap + Ai} \right)}}{2} - \frac{{\frac{Amix}{{\left( {Ap + Ai} \right)}}}}{2}} \right] \times 100 $$

Ap, Ai, and Amix denote the absorbance of pathogenic bacteria, selected strains, and their mixture after incubation.

### Cell culture conditions and treatments

The KB (ATCC CCL-17), OSCC (ATCC CRL-1628), fibroblast (ATCC CCL-127TM), and HUVEK (ATCC CRL-1730) cell lines were obtained from the Pastor Institute in Iran and cultured in Eagle’s Minimum Essential Medium (EMEM) from Sigma-Aldrich. The culture medium was supplemented with 10% fetal bovine serum (FBS, Gibco) and 1% penicillin–streptomycin solution (PAN-Biotech) and maintained at 37 °C with 5% CO_2_. The cells were seeded in 96-well plates at a density of 2000 cells per well and incubated for 24 h until reaching 80% confluence^6^.

Subsequently, the EMEM medium was replaced with bacterial secretions (200 µL, pH 7 without antibiotic solution), and the cells were further incubated for 24, 48, and 72 h. The efficacy of bacterial secretions was evaluated against untreated cells, as well as cells treated with MRS, anti-cancer drugs (doxorubicin and paclitaxel), and a commercially available probiotic strain (*Lactobacillus casei* subsp. *casei* PTCC 1608), which served as control groups.

### Cytotoxic test on cancer and normal cell lines

To conduct the MTT assay, cells were seeded at a density of 2 × 10^4^ cells/mL onto 96-well plates and incubated for 24 h. Bacterial secretions were obtained by centrifugation at 8000×*g* for 20 min at 4 °C. After adjusting the pH to 6.5, bacterial extracted metabolites at different concentrations (ranging from 1 to 25 µg/mL) were added to the cells and further incubated at 37 °C for 24, 48, and 72 h. Subsequently, 20 μL of MTT solution (5 mg/mL) was added to each well and incubated for an additional 3 h. The medium was removed, and the formazan crystals were dissolved in 150 μL of dimethyl sulfoxide. The absorbance was then measured using an ELISA reader at 570–630 nm.

In this experiment, untreated cells, cells treated with MRS, *L. casei* subsp. *casei* PTCC 1608, doxorubicin (0.7 µg/mL), and paclitaxel (0.5 µg/mL) were used as negative and positive controls for comparison. Additionally, to determine whether the anti-cancer metabolites have protein structures, pronase (Roche Applied Science) at a concentration of 1 mg/mL was added to the bacterial supernatants. They were incubated in the incubator for 30 min at 37 °C. The cell viability percentage for each sample was calculated using the following equation^6^:

$$ Cell\;viability\left( \% \right) = \frac{{\left( {\left[ A \right] sanple - \left[ A \right] blank} \right)}}{{\left( {\left[ A \right] control - \left[ A \right] blank} \right)}} \times 100 $$

### Fluorescent staining

To conduct the dual acridine orange/ethidium bromide (AO/EB) staining, cells were seeded in 24-well plates and treated with the most effective concentration of the bacterial extracts for 12 h. The cells were then harvested and washed three times with cold PBS. Subsequently, they were suspended in a mixture of acridine orange and ethidium bromide (1:100 mg/mL) with a volume of 100 μL. Following this, 10 μL of the stained cell suspension was placed on a slide, and both normal intact and apoptotic cells were visualized and analyzed under a fluorescent microscope (Bioteck, CellCytaion, USA).

### Apoptotic gene expression

SMAC and SURVIVIN are apoptotic genes that were studied in this study because they play a crucial role in the regulation of apoptosis, a process of programmed cell death of cancers. The cancer cells were seeded in a 6-well plate (2 × 10^5^ cells/well) and allowed to incubate for 24 h. Subsequently, the cells were treated with Y33 and pronase-treated Y33 secretion (20 µg/mL, 72 h). The control groups included untreated cancer cell lines and cells treated with an in-market probiotic strain (*L. casei* subsp. *casei* PTCC 1608). Total RNA extraction was performed from both treated and untreated cancer cell lines using TRIzol reagent according to the manufacturer’s instructions (Invitrogen, USA). A volume of 10 μL was obtained by adding 3 μL of Diethyl pyrocarbonate (DEPC) water. The extracted total RNA was evaluated for quantity and quality using the NanoDrop device and electrophoresis on a 1% agarose gel^6^.

The cDNA synthesis was performed following the manufacturer’s instructions. Quantitative real-time PCR (qRT-PCR) was employed to evaluate the mRNA expression of apoptotic-related genes (SMAC and SURVIVIN), with the β-actin gene serving as the endogenous control. The primers utilized in this study were as follows: β-actin (ACTB) gene: F-AGAGCTACGAGCTGCCTGAC and R-AGCACTGTGTTGGCGTACAG; SMAC (DIABLO) gene: F-CAGAGGAGGAAGATGAAGTGTG and R-GCGGTTATAGAGGCCTGATCTG; SURVIVIN (BIRC5) gene: F-CCCTTTCTCAAGGACCACCG and R-GTTCCTCTATGGGGTCGTCA. The PCR reaction mixture consisted of cDNA, PCR pre-mix, reverse primers, and forward primers (1:5:0.5:0.5). The PCR program comprised initial denaturation at 94 °C for 1 min, denaturation for 40 cycles at 94 °C for 20 s, annealing for 40 cycles at 59 °C for 30 s, and extension for 40 cycles at 72 °C for 30 s.

#### DNA extraction and molecular identification

The total DNA of the isolates from the cultures inoculated with a single colony was extracted using the method described by Nami et al.^10^. In this process, a single colony was re-cultured in MRS broth for 24 h at 37 °C, and 1.5 mL of the bacterial culture was then centrifuged at 10,000×*g* for 5 min. The supernatant was discarded, and the pellet was used to isolate the DNA. The extracted DNA was suspended in 50 µL of distilled water, and all the extracted genomic DNAs of the samples were checked and visualized via 0.8% agarose gel electrophoresis. Subsequently, the gel monitoring apparatus (Biometra, Gottingen, Germany) and spectrophotometric method were used to evaluate the quality and quantity of the extracted DNA, respectively.

To amplify the 16S rRNA gene (1500 bp) of the bacterial strains, a specific pair of primers (Hal6F/Hal6R) designed for *Lactobacillus* was used. The forward primer had the sequence 5′-AGAGTTTGATCMTGGCTCAG-3′, and the reverse primer had the sequence 5′-TACCTTGTTAGGACTTCACC-3′. The PCR protocol included an initial denaturation step at 95°C for 5 min, followed by 30 cycles of denaturation at 94 °C for 60 s, primer annealing at 57 °C for 60 s, and extension at 72 °C for 120 s. A final extension step at 72 °C for 10 min was added. The PCR products were sequenced by the Macrogen DNA Sequencing Service (Korea), and the resulting sequences were aligned using Clustal^8^.

#### Statistical analysis

The experiments were conducted in triplicate using a randomized design. Statistical analysis was performed using SPSS 18 software. Duncan’s test was employed at a significance level of 95% (*P* ≤ 0.05).

#### Ethics-statement

This study was reviewed and approved by Dr. Mahmood Reza Moradi (Chairman of the Academic/Regional Ethics Committee in Biomedical Research) and Dr. Reza Khodarahmi (Secretary of the Academic/Regional Ethics Committee in Biomedical Research) of Kermanshah University of Medical Sciences (Approval ID: IR.KUMS.REC.1400.106). All methods were performed in accordance with the relevant guidelines and regulations by including a statement in the methods section.

## Result and discussion

### Morphological analysis of *Lactobacillus* strains

Lactic acid bacteria (LAB) constitute a group of microorganisms renowned for their advantageous health effects and recognized as safe for consumption or incorporation into food products. The majority of LAB species fall within the *Lactobacillus* genus. Isolation of the chosen *Lactobacillus* strains was achieved through biochemical and morphological analysis on specific growth media (MRS). The bacterial colonies displayed a characteristic whitish to creamy hemispherical form, indicative of Lactobacilli. Subsequently, 21 Gram-positive and negative bacteria were isolated and cultured in an MRS medium under anaerobic conditions. These 21 isolates were further selected for detailed characterization, with their respective details presented in Table 1.

**Table 1:** Dairy origin, catalase test, gram staining, and survival rate of isolated *Lactobacillus* strains after 3–4 h of incubation at 0.3% bile salt, and pH 2.5–6.6

Survival rates = ([OD_600_ (3–4 h)/OD_600_ (0 h)] × 100)						
Isolated strains	Origin	Catalase test	Gram staining	Tolerance after 4 h at 0.3% bile	Tolerance after 3 h at pH 2.5	Tolerance after 3 h at pH 6.6
C5	Cheese	Negative	*Gram*-positive	85.43 ± 1.36^c^	81.38 ± 1.26^b^	126.30 ± 1.78^a^
C7a	Cheese	Negative	*Gram*-positive	56.73 ± 1.46^f^	51.73 ± 1.64^g^	124.92 ± 1.42^b^
C7b	Cheese	Negative	*Gram*-positive	16.98 ± 5.47^m^	18.28 ± 1.10^n^	106.19 ± 1.41^g^
C28	Cheese	Negative	*Gram*-positive	87.15 ± 2.08^b^	78.05 ± 2.38^c^	102.53 ± 1.14^i^
C30	Cheese	Negative	*Gram*-positive	16.24 ± 1.46^m^	12.68 ± 0.55^o^	92.28 ± 1.44^l^
C34	Cheese	Negative	*Gram*-positive	*30.38* ± *1.24*^k^	*28.78* ± *2.36*^l^	97.36 ± 0.82^k^
C45	Cheese	Negative	*Gram*-positive	*42.68* ± *1.44*^i^	*40.25* ± *1.66*^i^	99.62 ± 1.82^j^
M8	Milk	Negative	*Gram*-positive	43.44 ± 0.70^i^	35.43 ± 0.87^j^	127.41 ± 1.68^a^
M16	Milk	Negative	*Gram*-positive	*78.52* ± *1.37*^d^	*67.24* ± *1.55*^d^	110.62 ± 1.18^f^
M19	Milk	Negative	*Gram*-positive	23.34 ± 1.02^l^	11.47 ± 1.12^op^	100.41 ± 0.83^j^
M34	Milk	Negative	*Gram*-positive	*15.68* ± *0.18*^m^	*10.23* ± *0.76*^p^	88.44 ± 1.32^m^
M45	Milk	Negative	*Gram*-positive	*88.52* ± *1.48*^b^	*82.37* ± *0.48*^b^	118.80 ± 1.28^d^
M49	Milk	Negative	*Gram*-positive	58.32 ± 4.47^e^	57.21 ± 0.65^e^	115.89 ± 1.39^e^
Y6	yogurt	Negative	*Gram*-positive	49.10 ± 1.13^g^	42.61 ± 1.31^h^	106.00 ± 2.38^g^
Y13	yogurt	Negative	*Gram*-positive	45.75 ± 1.66^h^	32.68 ± 3.09^k^	102.18 ± 1.54^i^
Y21	yogurt	Negative	*Gram*-positive	23.12 ± 0.88^l^	21.50 ± 1.42^m^	99.20 ± 1.77^j^
Y25	yogurt	Negative	*Gram*-positive	*77.38* ± *1.08*^d^	*68.45* ± *2.02*^d^	106.58 ± 1.48^g^
Y27	yogurt	Negative	*Gram*-positive	59.60 ± 1.66^e^	54.78 ± 1.18^f^	111.77 ± 1.65^f^
Y33	yogurt	Negative	*Gram*-positive	118.80 ± 0.76^a^	98.58 ± 0.45^a^	118.66 ± 0.79^d^
Y44	yogurt	Negative	*Gram*-positive	*40.32* ± *0.68*^j^	*31.85* ± *0.78*^k^	105.40 ± 1.90l^g^
Y49	yogurt	Negative	*Gram*-positive	*22.70* ± *1.06*^l^	*18.36* ± *1.12*^n^	101.24 ± 1.82^ij^

*Values are mean ± standard error of triplicates. ^a–p^Means in the same column with different lowercase letters differed significantly (P < 0.05). Significant values are in italics.

### Simulated digestive conditions tolerance test

In addition to biochemical and morphological analysis, the isolated *Lactobacillus* strains underwent further characterization, assessing their resistance to harsh conditions through various in vitro tests. These tests included exposure to adjusted PBS with high bile salt and low pH, as previously described by Park et al.^11^. Additionally, the strains were evaluated using a dynamic GI model, replicating gastric and intestinal growth conditions, following the methodologies of Fernandez et al.^12^. The selection of strains resilient to these harsh conditions is crucial for ensuring their survival and efficacy in the human digestive system.

The viability of the selected strains was further assessed using a dynamic GI model, simulating the conditions of the human digestive system. The introduction of the four selected strains into the model, mimicking stomach and small intestine requirements, demonstrated that M45 and Y33, in particular, exhibited higher resistance to harsh GI conditions, maintaining their viability in the simulated conditions. These findings suggest that these strains could serve as effective probiotic candidates for future incorporation into food products.

The study’s findings, indicating the resilience of strains M45 and Y33 to harsh gastrointestinal conditions in a dynamic GI model, provide valuable insights into potential probiotic candidates for food product incorporation. The ability of these strains to withstand simulated stomach and small intestine requirements is noteworthy, as it reflects their potential to survive and exert beneficial effects in the human digestive system. Several recent studies have emphasized the importance of strain-specific characteristics in determining probiotic efficacy. For instance, research by Wolfe et al.^13^ demonstrated that resistance to gastric acid and bile salts is crucial for probiotic survival in the GI tract. Moreover, a meta-analysis by Shi et al.^14^ highlighted the significance of robustness in probiotic strains, especially in acidic environments, which aligns with the observed resilience of M45 and Y33 in the simulated stomach conditions of the study.

Furthermore, recent advancements in probiotic research have explored innovative delivery methods and encapsulation techniques to enhance the survival and effectiveness of probiotic strains in the GI tract^15^. Considering these developments, the potential of M45 and Y33 as probiotic candidates gains significance, as their natural resistance to harsh GI conditions could complement emerging delivery strategies. In conclusion, the current study’s results align with recent research trends emphasizing the resilience of probiotic strains to harsh GI conditions. The potential of M45 and Y33 as effective probiotic candidates is supported by their ability to maintain viability in simulated digestive conditions. Nevertheless, further in vivo studies and consideration of recent research developments will be essential to solidify the practical application of these findings in the field of probiotics.

Moreover, all four selected isolates demonstrated good viability under intestinal conditions, with stable Log CFU/mL amounts after 2 h of incubation (Table 2). These results indicate the potential of the selected *Lactobacillus* strains to survive and tolerate the harsh conditions of the human GI tract, particularly in the intestinal environment.

**Table 2:** Re-screening and survival rate (%) of isolated *Lactobacillus* strains under gastric and intestinal digestive conditions.

Isolated strains	Final counts (Log CFU/mL) after incubation in gastric conditions				Final counts (Log CFU/mL) after incubation at intestinal conditions				
	0 h	1 h	2 h	SR (%)	0 h	1 h	2 h	3 h	SR (%)
C5	9.318 ± 0.16	8.254 ± 0.14	3.727 ± 0.10	40^c^	9.738 ± 0.22	9.048 ± 0.14	4.728 ± 0.12	4.090 ± 0.14	42^c^
C28	9.514 ± 0.16	8.619 ± 0.12	4.471 ± 0.10	47^b^	9.323 ± 0.16	8.724 ± 0.11	5.583 ± 0.14	4.941 ± 0.22	53^b^
M45	9.713 ± 0.12	8.238 ± 0.15	1.748 ± 0.16	18^d^	9.518 ± 0.12	8.178 ± 0.16	4.208 ± 0.14	3.141 ± 0.15	33^d^
Y33	9.012 ± 0.14	8.818 ± 0.17	6.579 ± 0.10	73^a^	9.624 ± 0.17	9.518 ± 0.15	7.742 ± 0.18	7.507 ± 0.11	78^a^
Con	9.131 ± 0.18	8.521 ± 0.23	6.392 ± 0.19	70^a^	9.252 ± 0.24	8.125 ± 0.24	7.351 ± 0.19	6.107 ± 0.24	66^ab^

*Values followed by the same letters are not significantly different (*P* < 0.05). Statistical analysis of each formulation was done separately. SR: Survival Rate. Con: *Lactobacillus casei* subsp*. casei* PTCC 1608.

### Anti-pathogen activities

Numerous studies have highlighted the potential of specific probiotic strains to exhibit anti-infective properties against intestinal microbes^7–9^. This anti-pathogenic activity against harmful bacteria underscores the importance of carefully considering probiotics in promoting health^16^. Probiotic strains designated as accepted probiotics are expected to demonstrate antimicrobial activities against both Gram-positive and Gram-negative pathogenic microorganisms.

The antagonistic activities of four isolated strains against eleven pathogens were evaluated under different pH conditions (neutral and natural pH) and treatments (catalase and proteinase K). The results, as presented in Table 3, reveal that the Y33 strain exhibited significant anti-pathogenic activity, inhibiting the growth of all tested pathogens. Additionally, C5 demonstrated a moderate level of antagonistic activity, inhibiting the growth of *S. aureus* and K. pneumonia.

**Table 3:** The inhibitory effect of isolated *Lactobacillus* strains against pathogens.

Pathogens	Diameter of inhibition zone (mm)				
	C5	C28	M45	Y33	Con
*S. sanguinis*	0.0 ± 0.0^b^	0.0 ± 0.0^a^	0.0 ± 0.0^a^	15.7 ± 0.3^a^	0.0 ± 0.0^b^
*S. salivarius*	0.0 ± 0.0^b^	0.0 ± 0.0^a^	0.0 ± 0.0^a^	16.5 ± 0.2^a^	0.0 ± 0.0^b^
*S. sobrinus*	0.0 ± 0.0^b^	0.0 ± 0.0^a^	0.0 ± 0.0^a^	15.7 ± 0.2^a^	0.0 ± 0.0^b^
*Y. enterocolitica*	0.0 ± 0.0^b^	0.0 ± 0.0^a^	0.0 ± 0.0^a^	15.2 ± 0.5^a^	0.0 ± 0.0^b^
*S. mutans*	0.0 ± 0.0^b^	0.0 ± 0.0^a^	0.0 ± 0.0^a^	14.2 ± 0.3^a^	0.0 ± 0.0^b^
*P. aeruginosa*	0.0 ± 0.0^b^	0.0 ± 0.0^a^	0.0 ± 0.0^a^	16.5 ± 0.4^a^	0.0 ± 0.0^b^
*S. aureus*	8.2 ± 0.4^a^	0.0 ± 0.0^a^	0.0 ± 0.0^a^	15.5 ± 0.2^a^	13.4 ± 0.3^a^
*B. subtilis*	0.0 ± 0.0^b^	0.0 ± 0.0^a^	0.0 ± 0.0^aa^	14.6 ± 0.2^a^	11.4 ± 0.2^a^
*L. monocytogenes*	0.0 ± 0.0^b^	0.0 ± 0.0^a^	0.0 ± 0.0^a^	14.7 ± 0.1^a^	9.2 ± 0.3^a^
*K. pneumoniae*	6.3 ± 0.7^a^	0.0 ± 0.0^a^	0.0 ± 0.0^a^	16.7 ± 0.3^a^	0.0 ± 0.0^b^
*S. flexneri*	0.0 ± 0.0^b^	0.0 ± 0.0^a^	0.0 ± 0.0^a^	15.8 ± 0.1^a^	0.0 ± 0.0^b^

ATCC: American Type Culture Collection, Virginia, USA. PTCC: Persian Type Culture Collection, Tehran, Iran. *Streptococcus sanguinis* PTCC 1449, *Streptococcus salivarius* PTCC 1448, *Streptococcus sobrinus* PTCC 1601, *Yersinia enterocolitica* ATCC 23715, *Streptococcus mutans* PTCC 1683, *Pseudomonas aeruginosa* PTCC 1181, *Staphylococcus aureus* ATCC 25923, *Bacillus subtilis* ATCC 19652, *Listeria monocytogenes* ATCC 13932, *Klebsiella pneumoniae* PTCC 1053, and *Shigella flexneri* PTCC 1234.

*Values are mean ± standard error of triplicates. ^a–d^Means in the same row with different lowercase letters differed significantly (*P* < 0.05). Con: *Lactobacillus casei* subsp*. casei* PTCC 1608.

Adjusting the pH to 6.8 revealed that the anti-pathogenic activities of Y33 and C5 were attributed to acid production against certain pathogens. Furthermore, after treatment with catalase enzyme, Y33’s inhibitory effect on *B. subtilis* and *L. monocytogenes*, as well as C5’s inhibition of *S. aureus*, indicated hydrogen peroxide production as a mechanism. Notably, after treatment with proteinase K enzyme, the absence of inhibitory halos for Y33 against *Y. enterocolitica* and *P. aeruginosa* confirmed the proteinaceous nature of the bacterial extracts against these pathogens.

The results of the study, detailing the antagonistic activities of four isolated strains against eleven pathogens under different conditions and treatments, provide valuable insights into the potential mechanisms behind their anti-pathogenic effects.

The Y33 strain emerges as a particularly promising candidate, demonstrating significant anti-pathogenic activity by inhibiting the growth of all tested pathogens. This broad-spectrum activity is of considerable interest, especially in the context of addressing multiple pathogenic challenges. Similar findings have been observed in recent studies, such as the work by Kim et al.^17^, where certain bacterial strains exhibited broad-spectrum antimicrobial effects due to the production of specific metabolites.

C5, on the other hand, exhibits a moderate level of antagonistic activity, specifically inhibiting the growth of *S. aureus* and *K. pneumonia*. This targeted inhibition may be linked to strain-specific characteristics or the production of antimicrobial compounds effective against these particular pathogens. A research by Campana et al.^18^ highlighted the importance of understanding strain-specific antimicrobial activities for the development of effective probiotics.

The adjustment of pH to 6.8 unveils the role of acid production as a mechanism behind the anti-pathogenic activities of Y33 and C5 against certain pathogens. Acidic conditions have been recognized as a natural defense mechanism against microbial growth, and the ability of these strains to exploit this mechanism is consistent with the findings of Guan and Liu^19^ in their exploration of acid-mediated microbial inhibition.

Treatment with catalase enzyme reveals hydrogen peroxide production as a contributing mechanism to the inhibitory effects. Y33’s inhibition of *B. subtilis* and *L. monocytogenes*, as well as C5’s inhibition of *S. aureus*, indicates the involvement of hydrogen peroxide. This aligns with the well-established antimicrobial properties of hydrogen peroxide, as discussed in studies by Khurshid et al.^20^ and Linley et al.^21^.

The use of proteinase K enzyme further elucidates the nature of the bacterial extracts, confirming the proteinaceous nature of Y33’s inhibitory effects against *Y. enterocolitica* and *P. aeruginosa*. Similar protein-based antagonistic mechanisms have been reported in studies by Rodrigues et al.^22^, emphasizing the diversity of antimicrobial compounds produced by different bacterial strains.

In conclusion, the study’s results provide comprehensive insights into the antagonistic activities of Y33 and C5 against a variety of pathogens, highlighting the involvement of acid production, hydrogen peroxide, and proteinaceous compounds as potential mechanisms. These findings align with and contribute to the broader body of research on bacterial antagonism and provide a foundation for the development of antimicrobial strategies or probiotics with targeted effects against specific pathogens.

### Antibiotics sensitivities

The genetic structure of probiotic bacteria inherently contains elements that confer resistance to different antibiotics. However, the overuse of antibiotics has led to the emergence of new antibiotic-resistant genes in probiotics, which can potentially be transferred to other microorganisms in the digestive tract, including harmful pathogens. This raises concerns about the development of antibiotic resistance within the community^23^. Hence, evaluating the susceptibility of probiotics to antibiotics is a crucial factor in their selection.

The susceptibility of *Lactobacillus* strains to nine clinically essential and widely used antibiotics is detailed in Table 4. The results indicate that all four isolates were resistant to azithromycin and trimethoprim-sulfamethoxazole. However, the strains showed low resistance to other antibiotics. Notably, C28 and Y33 exhibited the best results, being susceptible or semi-susceptible to five antibiotics.

**Table 4:** Antibiotic susceptibility profiles of isolated *Lactobacillus* strains.

Isolated Strains	Antibiotics								
	CFM	AZM	AMX	D	SXT	CP	CN	AMC	V
C5	0.00 ± 0.00^b^	0.00 ± 0.00^a^	0.00 ± 0.00^c^	14.68 ± 1.18^d^	0.00 ± 0.00^a^	16.47 ± 0.62^b^	10.52 ± 1.26^d^	18.58 ± 0.38^d^	0.00 ± 0.00^b^
C28	16.44 ± 0.72^a^	0.00 ± 0.00^a^	0.00 ± 0.00^c^	46.24 ± 0.66^a^	0.00 ± 0.00^a^	0.00 ± 0.00^c^	31.84 ± 0.40^a^	32.70 ± 1.04^a^	20.14 ± 0.46^a^
M45	0.00 ± 0.00^b^	0.00 ± 0.00^a^	20.60 ± 0.33^b^	31.52 ± 1.22^c^	0.00 ± 0.00^a^	0.00 ± 0.00^c^	15.30 ± 1.48^b^	24.92 ± 0.74^c^	0.00 ± 0.00^b^
Y33	0.00 ± 0.00^b^	0.00 ± 0.00^a^	25.28 ± 0.66^a^	42.48 ± 0.84^b^	0.00 ± 0.00^a^	20.16 ± 0.27^a^	12.62 ± 0.55^c^	28.90 ± 0.48^b^	0.00 ± 0.00^b^

CFM, cefixime; AZM, azithromycin; AMX, amoxicillin; D, doxycycline; SXT, trimethoprim sulfamethoxazole; CP, ciprofloxacin; CN, cephalexin; AMC, amoxicillin–clavulanic acid; V, vancomycin. Cefixime results based on R ≤ 15 mm; I: 16–18 mm; S ≥ 19 mm. Azithromycin results based on R ≤ 13 mm; I: 14–17 mm; S ≥ 18 mm. Amoxicillin results based on R ≤ 18 mm; I: 19–21 mm; S ≥ 22 mm. Doxycycline results based on R ≤ 10 mm; I: 11–13 mm; S ≥ 14 mm. Trimethoprim sulfamethoxazole results based on R ≤ 25 mm; I: 26–29 mm; S ≥ 30 mm. Ciprofloxacin results based on R ≤ 15 mm; I: 16–20 mm; S ≥ 21 mm. Cephalexin results based on R ≤ 14 mm; I: 15–17 mm; S ≥ 18 mm. Amoxicillin–clavulanic acid results based on R ≤ 13 mm; I: 14–17 mm; S ≥ 18 mm. Vancomycin results based on R ≤ 14 mm; I: 15–16 mm; S ≥ 17 mm. Performance Standards for Antimicrobial Susceptibility Testing, from Clinical and Laboratory Standards Institute, Twenty-Third Informational Supplement, Wayne, PA (CLSI 2013).

*Values are mean ± standard error of triplicates. ^a–e^Means in the same row with different lowercase letters differed significantly (P < 0.05).

Understanding the susceptibility of probiotic bacteria to antibiotics is vital for evaluating their safety and potential impact on the gut microbiota. The observation that all four isolates were resistant to azithromycin and trimethoprim-sulfamethoxazole raises concerns, as these antibiotics are widely used for various clinical indications. This resistance pattern aligns with findings reported in recent studies, such as the work by Anisimova et al.^24^, where increased resistance of *Lactobacillus* strains to macrolides and sulfonamides was documented.

However, the strains exhibited low resistance to the remaining antibiotics, suggesting a relatively favorable susceptibility profile. Notably, C28 and Y33 stood out as the most promising strains, being either susceptible or semi-susceptible to five antibiotics. This is an encouraging finding, as it indicates that these strains may have a more favorable antibiotic resistance profile compared to the other isolates. Recent studies, such as the comprehensive review by Aghamohammad and Rohani^25^ have highlighted the need for continuous monitoring of antibiotic resistance in probiotics, given its potential implications for both human health and the development of antibiotic-resistant strains.

The resistance patterns observed in this study may be influenced by various factors, including the specific *Lactobacillus* strains studied, their origin, and the geographical distribution. Comparisons with global antibiotic resistance trends in probiotic strains, as discussed by Zyoud et al.^26^, would contribute to a more comprehensive understanding of the findings. It’s essential to note that antibiotic resistance in probiotic bacteria can be strain-specific, and the observed resistance patterns should be interpreted in the context of the intended use of these strains as probiotics.

In conclusion, the susceptibility results of *Lactobacillus* strains to nine clinically essential antibiotics underscore the importance of monitoring antibiotic resistance in probiotic bacteria. The observed resistance patterns, especially the resistance to azithromycin and trimethoprim-sulfamethoxazole, warrant further investigation and consideration of strain-specific characteristics. The favorable susceptibility profile of C28 and Y33 to multiple antibiotics is a positive indication, but ongoing surveillance and adherence to safety guidelines are crucial in the development and use of probiotics.

### Probiotic characterization

The evaluation of probiotics’ adhesion capacity to the hydrophobic phase is crucial for assessing their ability to adhere to the intestinal mucosa and prevent the adhesion of pathogens to the digestive tract^27,28^. The surface hydrophobicity results, presented in Table 5, indicate that isolate Y33 demonstrated significantly higher hydrophobic rates (62%) compared to the other tested isolates. The variation in surface hydrophobicity among *Lactobacillus* strains is mainly attributed to differences in the production of surface proteins^4^.

**Table 5:** Surface hydrophobicity (%), biofilm formation, cholesterol uptake (%), hemolytic activity, auto-aggregation (%), and co-aggregation (%) of isolated *Lactobacillus* strains.

Strains	Hydrophobicity (%)	Biofilm formation	Cholesterol removal (%)	Hemolysis	Auto-aggregation (%)	Co-aggregation		
						*E. coli* (PTCC 1276)	*L. monocytogenes* (ATCC 13932)	*B. subtilis* (ATCC 19652)
C5	25 ± 0.8^c^	22 ± 1.8^b^	18 ± 0.4^d^	γ	23 ± 1.6^c^	19.2 ± 1.34^b^	18.6 ± 1.52^c^	15.2 ± 1.48^b^
C28	31 ± 1.2^b^	23 ± 0.4^b^	21 ± 1.7^c^	γ	26 ± 1.4^b^	16.5 ± 1.75^c^	22.1 ± 1.35^b^	09.4 ± 1.12^d^
M45	09 ± 1.4^d^	12 ± 1.6^c^	26 ± 1.8^b^	α	08 ± 0.8^d^	12.4 ± 1.80^d^	14.7 ± 1.32^d^	12.6 ± 1.88^c^
Y33	62 ± 2.1^a^	52 ± 0.4^a^	42 ± 1.3^a^	γ	57 ± 2.3^a^	56.2 ± 1.35^a^	42.1 ± 1.67^a^	67.2 ± 1.98^a^

Values shown are means ± standard deviations (n = 3).

*Values followed by the same letters are not significantly different (P < 0.05). Statistical analysis of each formulation was done separately.

Adherence to intestinal epithelial cells is another vital characteristic of probiotics, and the results show that strain Y33 exhibited the most robust adhesion to human intestinal Caco-2 cells (52%). On the other hand, strains M45, C5, and C28 displayed weaker adherence abilities. The ability to adhere to intestinal epithelial cells is essential for probiotics to colonize the gut effectively^7^.

The cholesterol elimination ability of the isolates was also assessed, and isolate Y33 exhibited the highest cholesterol uptake, absorbing more than 42% of cholesterol after 20 h of incubation. The mechanisms behind cholesterol reduction by probiotics include enzymatic conversion, incorporation into the cell wall, and disruption of cholesterol micelle formation in the intestine. The results suggest that Y33 may have potential cholesterol-lowering effects.

The hemolytic properties of the isolates were examined, and only one strain (M45) exhibited α-hemolytic activity, while the others (C5, C28, and Y33) showed γ-hemolytic properties. It’s important to note that the lack of blood cell hemolysis (γ-hemolytic) does not necessarily indicate the safety of probiotic strains.

Auto-aggregation and co-aggregation are crucial mechanisms for probiotics in preventing surface colonization by pathogens^8,9,16^. The auto-aggregation assay revealed that isolate Y33 demonstrated the highest auto-aggregation percentage (57%). In terms of co-aggregation, all tested isolates showed the ability to aggregate with different pathogens, with Y33 exhibiting the highest co-aggregation percentages with *E. coli*, *L. monocytogenes*, and *B. subtilis*.

The observed antimicrobial activities are associated with the aggregation capability, and Y33 showed the highest antibacterial and aggregation abilities among the tested isolates. This can be attributed to the complex interaction between surface molecules, secreted factors, and proteins. The close contact between probiotics and pathogens during aggregation is crucial for the release of antimicrobial substances, leading to the suppression of pathogens^16,29^. Overall, these findings highlight the potential probiotic properties of isolate Y33, including its adhesion capacity, cholesterol-lowering ability, and antimicrobial activities.

### Pre-screening cytotoxic test on KB cells

The research on the anti-cancer properties of selected *Lactobacillus* isolates, including C5, C28, M45, and Y33, adds to the growing body of evidence highlighting the potential health-promoting properties of probiotics. Previous studies have demonstrated therapeutic effects and a protective role of *Lactobacillus* strains against various cancers, such as liver, breast, gastric, bladder, and colon cancers.

**Figure 1:**
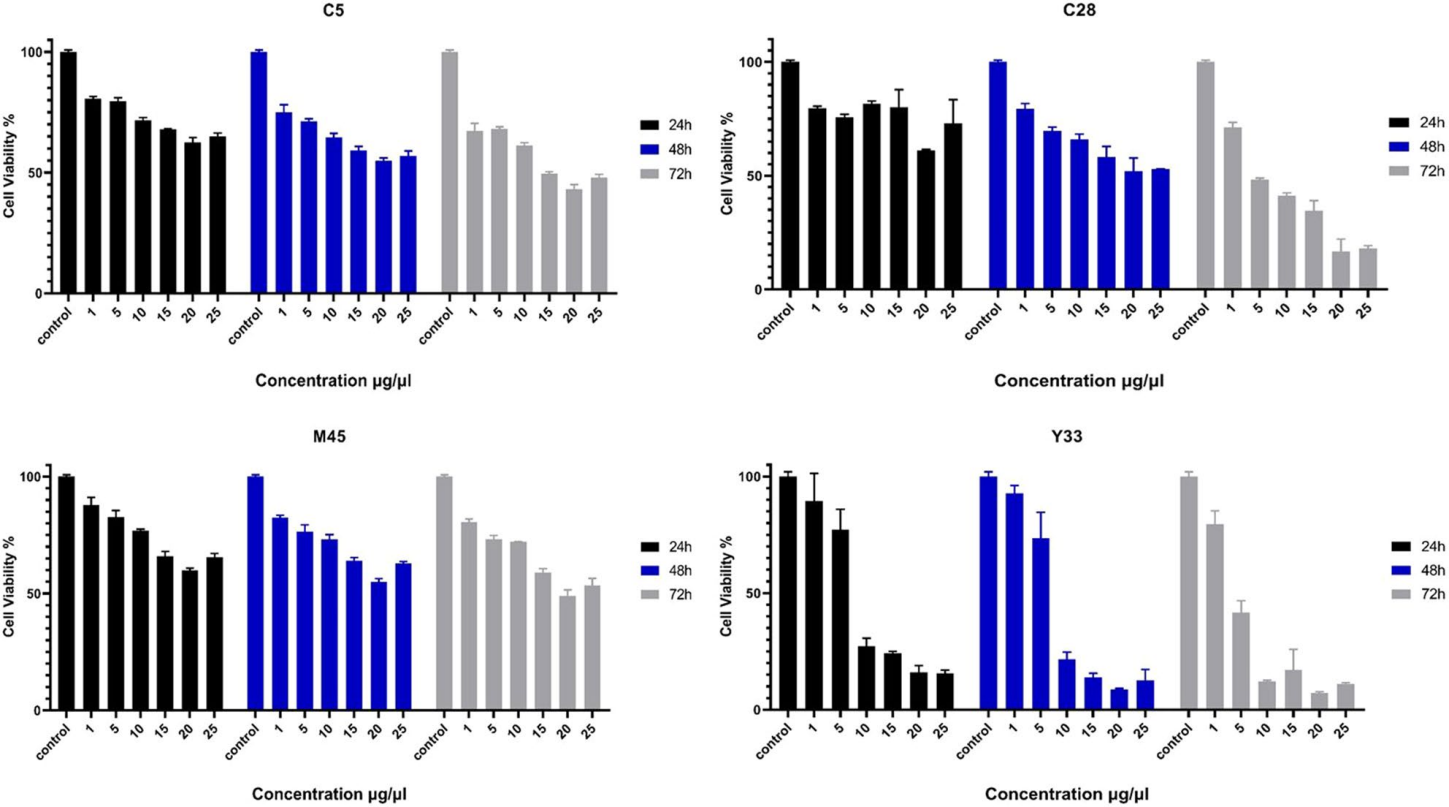
The cytotoxic effects of C5, C28, M45, and **Y33** secretions (1–25 µg/mL) on KB cancer cell line after 24 h, 48 h, and 72 h incubation. Each bar represents the mean ± SE of six replicates. Error bares represent standard deviation of each mean. Control: untreated cancer cell line.

In this study, in vitro methods were employed to evaluate the anti-cancer properties of the selected *Lactobacillus* isolates. A pre-screening test was conducted to determine the most effective *Lactobacillus* secretions by assessing different concentrations (0, 1, 5, 10, 15, 20, and 25 µg/mL) after 24, 48, and 72 h. The results, as shown in Fig. 1 and Table 6, indicate that the cytotoxic effects of the extracted metabolites on KB cancer cell lines were dose and time-dependent.

**Table 6:** The IC_50_ values of C5, C28, M45, and Y33 secretions (1–25 µg/mL) on KB cancer cell line after 24 h, 48 h, and 72 h incubation.

Extractions	KB		
	24 h	48 h	72 h
C5	ND	ND	14.657 µg/µL
C28	ND	ND	4.281 µg/µl
M45	ND	ND	19.625 µg/µL
Y33	7.723 µg/µl	6.944 µg/µl	3.213 µg/µl

*ND, not detected value.

Specifically, the extracted metabolite of the Y33 strain displayed significantly lower cell viability (at the 0.05 level) at the concentration of 20 µg/mL compared to other concentrations (0, 1, 5, 10, 15, and 25 µg/mL). Therefore, 20 µg/mL was chosen as the effective concentration. Additionally, after 72 h of incubation, the lowest cell viabilities and the highest anti-cancer activities were observed. The cell viabilities at this time point were significantly lower than at 24 and 48 h, being under 26% (Fig. 1).

These findings suggest that the extracted metabolites from the Y33 strain have a potent anti-cancer effect on KB cancer cell lines, and the concentration of 20 µg/mL, after 72 h of incubation, demonstrated the most significant impact. The time-dependent cytotoxicity indicates that prolonged exposure enhances the anti-cancer activity. Further investigations and characterization of the active compounds responsible for these anti-cancer effects would contribute to a better understanding of the potential therapeutic applications of these probiotic strains.

### Cytotoxic test on different cancer and normal cell lines

The evaluation of the 20 µg/mL extracted metabolite of the Y33 strain after 24, 48, and 72 h of incubation on oral cancer cell lines (KB and OSCC) and normal cell lines (fibroblast and HUVEK), in comparison with untreated cells, MRS-treated cells, and drug-treated cell lines (doxorubicin and paclitaxel) was performed using the MTT assay^7^.

**Figure 2:**
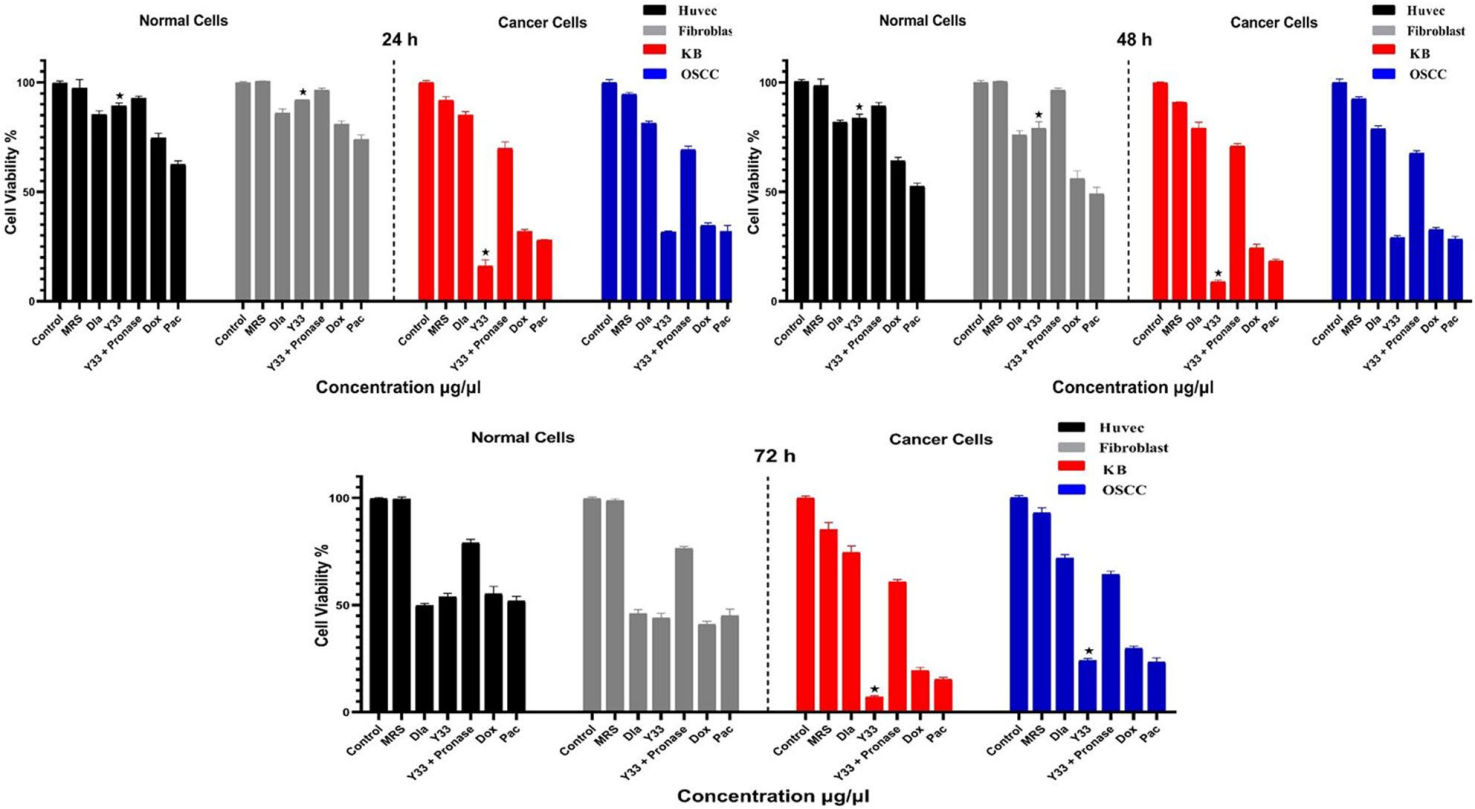
The cytotoxic effects of Y33 and pronase treated Y33 secretion (20 µg/mL) on oral cancer (KB and OSCC) and normal (fibroblast and HUVEK) cell lines after 24 h, 48 h and, 72 h incubation. Each bar represents the mean ± SE of six replicates. Error bares represent standard deviation of each mean. Asterisks denote statistically significant differences at < 0.05 level as compared with positive controls (doxorubicin and paclitaxel). Control: untreated cancer cell line. MRS: MRS treated cell lines. Dla: treated cells with in-market probiotic strain (*Lactobacillus acidophilus* PTCC 1643). Y33 + Pronase: pronase treated Y33 secretion. Dox: treated cells with doxorubicin anticancer drug. Pac: treated cells with paclitaxel anticancer drug.

The results indicate that the Y33 probiotic strain displayed high anti-cancer activities on KB and OSCC cell lines. The mean differences in cancer cell viability for the Y33 strain and anti-cancer drugs (doxorubicin and paclitaxel) after 24, 48, and 72 h of incubation, compared with negative controls, were significantly low (lower than 36%) at the 0.05 level, demonstrating their anti-cancer activities. Furthermore, the cell viability for the Y33 strain, in comparison with doxorubicin and paclitaxel, on the KB cell line after 24, 48, and 72 h and on the OSCC cell line after 72 h of incubation, was significantly lower at the 0.05 level, indicating better anti-cancer activities (Fig. 2).

On the other hand, the mean differences for normal cells (fibroblast and HUVEK) treated with the Y33 strain, compared with doxorubicin and paclitaxel-treated cells after 24 and 48 h of incubation, were significantly higher at the 0.05 level and didn’t show any side effects. However, after 72 h of incubation, Y33, doxorubicin, and paclitaxel significantly inhibited rapidly dividing normal cell lines (Fig. 2). This suggests that Y33 strain’s secreted metabolites have been identified as safe and cost-effective anti-cancer agents. Unlike other anti-cancer agents that are highly cytotoxic to sensitive cell lines and tissues, Y33 strain’s metabolites did not exhibit adverse effects on normal cell lines. In fact, the Y33 strain had the highest cytotoxic effect on oral cancer cell lines among all tested cell lines. Moreover, after 72 h of incubation, the cytotoxicity of Y33 strain’s metabolites was observed to be more pronounced in cancer cells than in normal cells, highlighting the selectivity of toxicity effects on cancer cells during this incubation period (as depicted in Fig. 2).

The exact mechanisms for the inhibition and prevention of cancer cell lines by the extracted metabolites of probiotics are unclear. The mean differences for pronase-treated Y33 metabolite compared to typically removed metabolite and anti-cancer drugs (doxorubicin and paclitaxel) were significantly higher in KB and OSCC cancer cell lines after 24, 48, and 72 h. It demonstrated that effective proteins were vital in the cytotoxic effects of Y33 secretions (Fig. 2). On the other hand, the mean differences for pronase-treated Y33 metabolites compared to untreated metabolites were not significant after 24 and 48 h of incubation in normal cell lines. This proved the existence of other cytotoxic mechanisms, and the effective proteins were not cytotoxic factors on normal cell lines (Fig. 2). Using effective proteins, probiotics can reduce harmful and carcinogenic enzymes like β-glycosidase, β-glucuronidase, IO hydratase-dehydrogenase, nitroreductase, nitrate/nitrite reductase, and azoreductase. These findings are supported by existing research and indicate that effective proteins play a crucial role in the cytotoxic effects of isolated *Lactobacillus* strains.

The results highlighting the high anti-cancer activities of the Y33 probiotic strain on KB and OSCC cell lines are promising and suggest the potential utility of this strain in cancer therapeutics. The comparison of Y33’s performance with conventional anti-cancer drugs like doxorubicin and paclitaxel provides valuable insights into its efficacy.

The mean differences in cancer cell viability for Y33, doxorubicin, and paclitaxel, especially when compared with negative controls, being significantly low (lower than 36%) at various time points (24, 48, and 72 h), underscore the consistent and substantial anti-cancer activities of the Y33 strain. This aligns with the growing body of research exploring the anti-cancer properties of probiotic strains. Recent studies, such as the work by Wang et al. (2022), have demonstrated the ability of specific probiotic strains to induce anti-cancer effects through various mechanisms, including modulation of the immune system and production of bioactive compounds^30^.

Comparative analysis reveals that the cell viability for the Y33 strain is significantly lower than that of doxorubicin and paclitaxel on the KB cell line after 24, 48, and 72 h, as well as on the OSCC cell line after 72 h of incubation. This suggests that Y33 not only exhibits anti-cancer activities but also performs better than the conventional anti-cancer drugs in certain conditions. Such findings are noteworthy, as they point towards the potential of probiotic strains like Y33 as alternative or complementary therapies for cancer treatment. It’s crucial to acknowledge that the anti-cancer mechanisms of probiotic strains can be diverse, involving direct cytotoxic effects, immunomodulation, and production of bioactive molecules. The study by Sener et al.^28^ highlights the multifaceted roles of probiotics in cancer prevention and treatment.

While these results are promising, further research is warranted to elucidate the specific mechanisms through which Y33 exerts its anti-cancer effects. Additionally, in vivo studies and clinical trials are essential for validating the efficacy and safety of probiotic strains in human subjects.

In conclusion, the findings regarding the high anti-cancer activities of the Y33 probiotic strain, along with its superior performance compared to conventional anti-cancer drugs, contribute to the evolving landscape of probiotics in cancer research. These results align with recent studies emphasizing the potential of probiotic strains as anti-cancer agents. However, continued exploration and validation are necessary to establish the clinical relevance and therapeutic potential of Y33 in cancer treatment.

### Fluorescent staining

The type of cell death is crucial as it indicates the nature of the response by neighboring tissues to drug treatment. Necrosis leads to oxidative stress and the release of numerous pro-inflammatory cytokines, whereas apoptosis is a controlled process typically characterized by minimal loss of membrane integrity before secondary necrosis or a later stage^29^.

**Figure 3:**
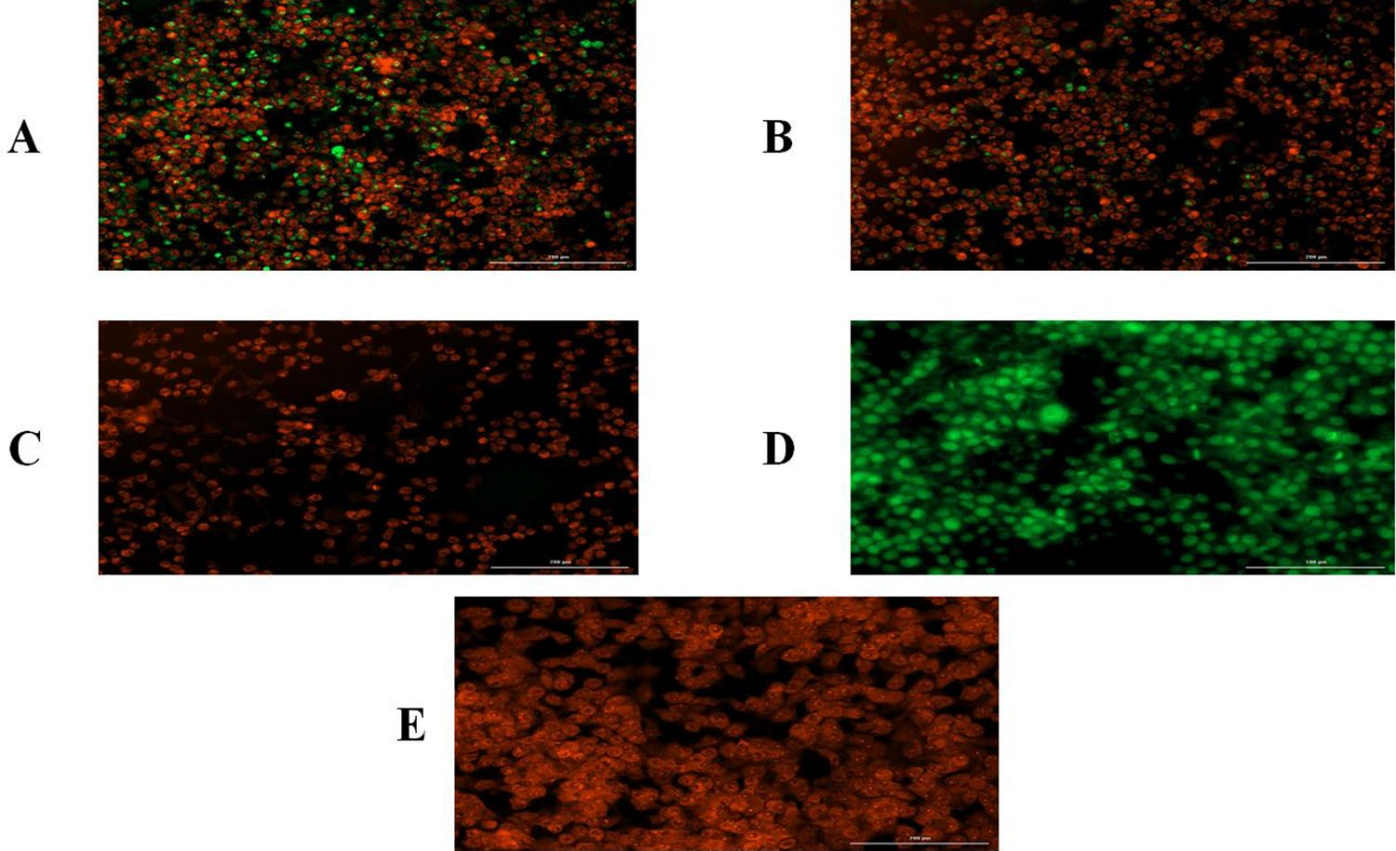
Dual acridine orange/ethidium bromide (AO/EB) fluorescent staining after incubation of Y33 secreted metabolite (20 µg/mL) on KB cancer cell line. Panels represent; (**A**) treated KB cancer cell line after 24-h incubation, (**B**) treated KB cancer cell line after 48-h incubation, (**C**) treated KB cancer cell line after 72-h incubation, (**D**) untreated KB cancer cell line as control, (**E**) treated KB cancer cell line after 72-h incubation with doxorubicin as positive control, green cells = normal intact cells, red cells = apoptotic cells.

After acridine orange/ethidium bromide (AO/EB) fluorescent staining, viable and apoptotic cells were determined. Viable cells were characterized as intact green cells, while orange-shrinking cells were represented as apoptotic cells. It was demonstrated that the mean number of apoptotic cells in treated KB cancer cell lines with Y33 secreted metabolite (20 µg/mL) was significantly higher at the 0.05 level compared to viable, necrotic cells, and spontaneous cell death in cell lines. Furthermore, the highest number of apoptotic cells was observed after 72 h of incubation (Fig. 3). According to the results, it was clear that Y33 secretion remarkably decreased viable cancer cells.

Moreover, cancer cells exhibited common properties of apoptotic cells, such as condensed chromatins, cell shrinkage, membrane blebbing, and the formation of apoptotic bodies. Nevertheless, the number of red cells did not increase, indicating that most of the dead cancer cells underwent apoptosis, not necrosis (Fig. 3). Recent research supports the use of AO/EB staining cells observed under the fluorescent microscope to distinguish apoptosis-related changes of cancer cell membranes within the apoptosis process^31^. These findings are consistent with the conclusion that the cytotoxicity of the isolated Y33 strain is generally mediated by apoptosis.

### Apoptotic gene expression

The key characteristics of cancer cells include resistance to apoptosis and uncontrolled proliferation. Compounds that promote apoptosis, such as probiotics, are considered potential anti-cancer agents. Apoptosis is a critical pathway in cancer development characterized by the inhibition of pre-apoptotic genes and the stimulation of anti-apoptotic genes. By targeting this pathway, probiotics and other therapeutic agents have the potential to induce apoptosis and inhibit cancer cell growth.

**Figure 4:**
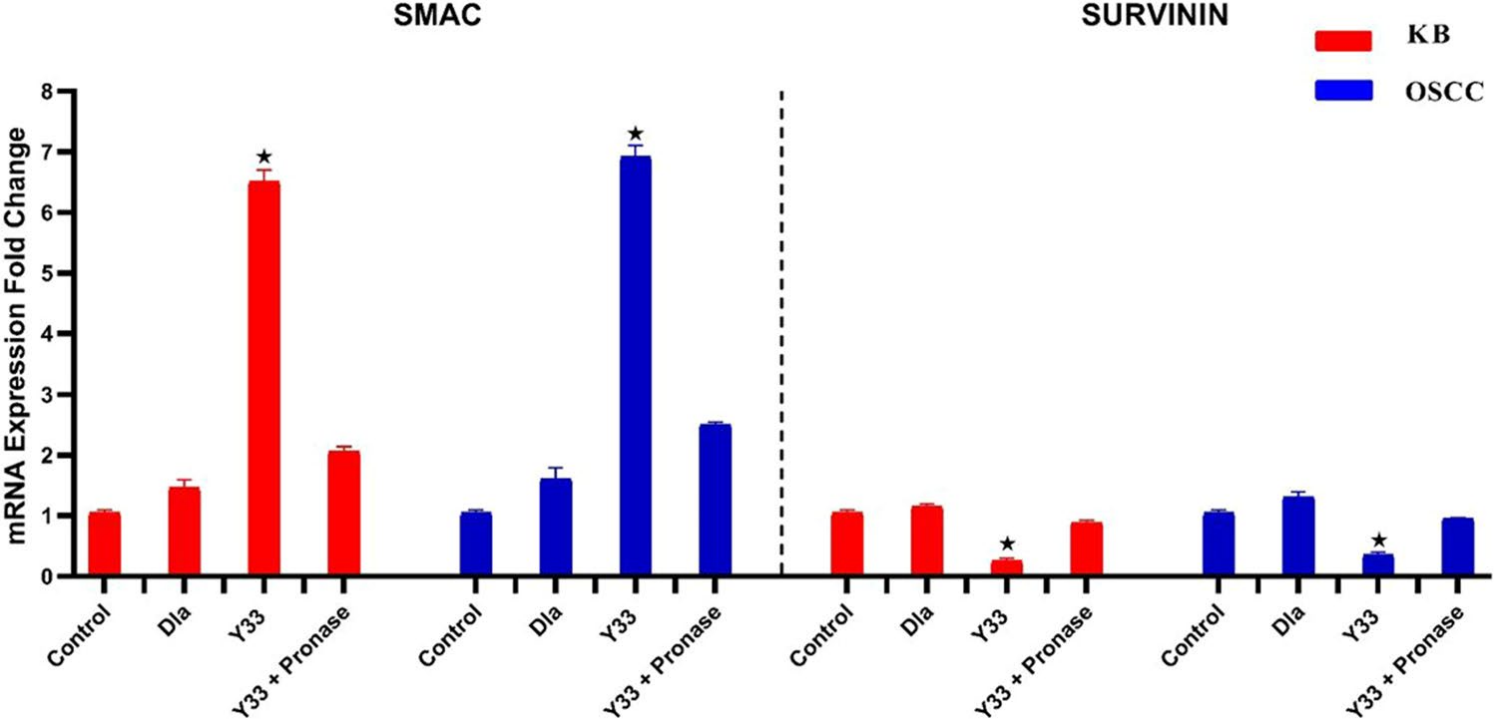
The mRNA expression of SMAC and SURVININ genes in KB and OSCC cancer cell lines treated with Y33 and pronase treated Y33 secretion (20 µg/mL) after 72 h incubation. Each bar represents the mean ± SE of six replicates. Error bares represent standard deviation of each mean. Asterisks denote statistically significant differences at < 0.05 level as compared with controls. Control: untreated cancer cell line. Dla: treated cells with in-market probiotic strain (*Lactobacillus casei* subsp*. casei* PTCC 1608). Y33 + Pronase: pronase treated Y33 secretion.

According to the findings, the Y33 probiotic strain extract exhibited a significant increase in mRNA expression of the SMAC gene in both KB and OSCC cancer cell lines. Simultaneously, there was a substantial reduction in the mRNA expression of the SURVIVIN gene in oral cancer cell lines treated with the Y33 secreted metabolites (refer to Fig. 4). These observations suggest the potential ability of the extracted metabolites of the Y33 probiotic strain to regulate apoptosis-related genes in oral cancer cells. In contrast, no significant change in mRNA expression of apoptotic genes were observed in untreated cancer cell lines and cells treated with an in-market probiotic strain (*Lactobacillus casei* subsp. *casei* PTCC 1608) as control groups. This indicates that there are other cell death mechanisms for the control groups, and apoptosis is not their main cytotoxic factor in oral cancer cell lines (Fig. 4). On the other hand, the mean differences for pronase-treated Y33 metabolite compared with normal Y33 extracted metabolite were significantly lower in the SMAC gene and considerably higher in the SURVIVIN gene. This demonstrates that effective proteins played a crucial role in the apoptotic effects of Y33 secretion (Fig. 4).

The results indicating a significant increase in mRNA expression of the SMAC gene and a substantial reduction in the mRNA expression of the SURVIVIN gene in both KB and OSCC cancer cell lines treated with the Y33 probiotic strain extract highlight the potential regulatory role of Y33 metabolites in apoptosis-related genes in oral cancer cells. These findings are intriguing and align with the emerging understanding of probiotics and their impact on molecular pathways in cancer research.

The up-regulation of the SMAC gene and down-regulation of the SURVIVIN gene suggest that the Y33 secreted metabolites may promote apoptosis in oral cancer cells. SMAC (Second Mitochondria-derived Activator of Caspases) is known to inhibit inhibitors of apoptosis proteins (IAPs) like SURVIVIN, leading to activation of caspases and induction of apoptosis. Similar observations have been reported in studies investigating the apoptotic effects of probiotics. For instance, the work by Ha et al.^32^ demonstrated the ability of certain probiotic strains to modulate apoptosis-related genes in colorectal cancer cells.

Importantly, the lack of significant changes in mRNA expression of apoptotic genes in untreated cancer cell lines and cells treated with an in-market probiotic strain (*Lactobacillus casei* subsp. *casei* PTCC 1608) as control groups indicates that apoptosis may not be the primary cytotoxic factor for these control groups in oral cancer cell lines. This underscores the specificity of the apoptotic effects observed with the Y33 strain and suggests that different probiotic strains may exert distinct effects on apoptosis-related pathways.

The comparison between pronase-treated Y33 metabolite and normal Y33 extracted metabolite, with significantly lower mean differences in the SMAC gene and considerably higher differences in the SURVIVIN gene, provides additional insights. This suggests that effective proteins, likely derived from Y33 secretion, play a crucial role in the apoptotic effects. The involvement of specific proteins in the apoptotic mechanisms induced by probiotic metabolites is consistent with the findings of studies like that of Martin et al.^33^, emphasizing the importance of understanding the proteinaceous nature of probiotic activities.

While these results are promising, it’s important to acknowledge the complexity of apoptotic pathways and the need for further mechanistic studies to elucidate the specific molecules and pathways involved. Additionally, in vivo studies and clinical trials are essential for translating these findings into practical applications.

In conclusion, the study’s findings regarding the modulation of apoptosis-related genes by the Y33 probiotic strain extract contribute to the growing body of evidence on the potential anti-cancer effects of probiotics. These results align with recent studies emphasizing the molecular mechanisms underlying the apoptotic effects of probiotic strains. However, continued research is necessary to unravel the specific components and pathways involved, as well as to validate the clinical relevance of these observations.

#### Molecular identification

To confirm the phenotypic characteristics of the selected bacterial strains, 16S rRNA gene sequencing was conducted. The amplification of the 16S rRNA genes confirmed that all four isolates chosen are members of the Lactobacillus genus.

Isolates C5 and C28 were identified as belonging to *Limosilactobacillus fermentum*, isolate M45 belonged to *Lactiplantibacillus pentosus*, and isolate Y33 belonged to *Lactiplantibacillus plantarum*. These sequences were submitted to the NCBI GeneBank with accession numbers OR230044, OR230046, OR230045, and OR230047, respectively.

The 16S rRNA sequencing method demonstrated high discriminatory power, providing easy-to-interpret patterns. This molecular technique is easy to perform, rapid, and reproducible. The results indicate that this method can be an effective and promising tool for identifying and discriminating dairy-associated Lactobacilli bacteria.

## Conclusion

In summary, the study identified *Lactiplantibacillus plantarum* Y33 as the most promising probiotic strain among the tested *Lactobacillus* strains. This strain demonstrated a high probiotic score, indicating its potential as a beneficial bacterium for human health. Additionally, the extracted bacteriocin from Y33, identified as a protein, exhibited significant cytotoxicity against KB and OSCC cancer cell lines, suggesting its potential as an anti-cancer agent for oral cancers. The findings propose that the Y33 strain has the potential to be utilized as a safe and effective probiotic supplement for enhancing human health and as a therapeutic agent for oral cancer treatment. However, further in vivo studies are necessary to validate the safety and efficacy of the Y33 strain and its extracted bacteriocin as potential probiotic and anti-cancer agents, respectively.

## Data Availability

The datasets generated and/or analysed during the current study are available in the NCBI GeneBank repository, accession numbers OR230044, OR230046, OR230045, and OR230047.
